# Evaluation of selected drying models of white sapote (*Casimiroa edulisL.*) slices as affected by drying methods and pre-drying treatments

**DOI:** 10.1016/j.heliyon.2024.e24440

**Published:** 2024-01-10

**Authors:** Tariku Workneh Daksa, Getachew Neme Tolesa

**Affiliations:** a Deaprtment of Food Technology and Process Engineering, Haramaya Institute of Technology, P.O. Box 138, Dire Dawa, Ethiopia; b Deaprtment of Food Science and Postharvest Technology, Haramaya Institute of Technology, P.O. Box 138, Dire Dawa, Ethiopia

**Keywords:** Dry matter, Drying methods, Drying models, Moisture ratio, Pre-drying treatments, White sapote

## Abstract

This investigation was performed on the drying modelling of white sapote slices that were influenced by drying methods and pre-drying treatments. The experimentation was performed by two factors: drying techniques and pre-drying treatments. The drying techniques included oven-, solar- and open sun-driers, whereas the pre-drying pretreatments were blanching, sodium metabisulfite, sodium-chloride and control. Behaviours of the drying were plotted with the moisture ratio vs time of drying, moisture content (% db) vs time of drying, and drying rate (g of water/100 g dm/hr) versus drying time (t). At the commencement of the drying progression, the drying rate was increased steadily and reduced through the advancement of drying model parameters a and n (empirical constants) and k (drying rate constant) were determined. Their combinations were optimised to suit the requirements of the minimal value of the sum of square deviation on the expected data values (model values). The models were evaluated with the regression coefficient (R^2^) and chi-square (χ^2^), resulting in values of 0.95–0.99 and 0.000132 to 0.00511, respectively. Henderson and Pabis's model demonstrated the highest coefficient R^2^ values (0.99) for oven-dried drying techniques. Also, the Page model gave the highest R^2^ value (0.99) for sodium metabisulphite pretreatments. It can be concluded that the Henderson and Pabis model is best suited for oven drying among the drying techniques. In contrast, the Page model best fits sodium metabisulphite among the pre-drying treatments. Hence, Henderson and Pabis, and Page models might describe the drying characteristics of the white sapote fruit slices based on respective drying methods and pre-drying treatments.

## 1 Introduction

Several research studies have reported the availability of white sapote fruit in different parts of Ethiopia. The habitation of white sapote in the Loma and Genabosa districts of Southern Ethiopia was found as home gardens [1]. The southern part of Ethiopia was reported to be the major utiliser of this crop (sapote) as food, and the people considered the use of the fruits of this plant as a stimulant [1]. Moreover, white sapote is available in Hawasa, the southern part of Ethiopia. The plant's local name is reported as the *Kasmire* and was determined to be indigenous to the reported place [2]. Moreover, the white sapote is a cultivated food plant in the home gardens of the town of Dheeraa in Arsi, Oromiya National Regional State, central Ethiopia [2]. The white sapote plants serve as the trees, which provide shade for coffee plants in cultivation and provide the fruits as the food source [3]. The white sapote plant is one of the food trees in the Southeastern Rift Valley of Ethiopia.

About 85 % of these plants are owned by the farmers and available in their house yards, and the remaining 15 % are growing in the Agro-forest system [3]. White sapote is an evergreen plant, but it is not intensively utilised as a food and feed source due to a lack of awareness among the public. Sapote is highly perishable. During peak harvesting seasons, the postharvest loss of white sapote fruit is 30–70 %; hence, the fruits are sold at low prices due to inadequate preservation techniques [4]. Therefore, value-addition processing is necessary to expand nutritional input and the market of white sapote fruit to avail it during off-seasons [5]). The white sapote fruit is an economically and socially important crop. Hence, it is an important fruit for commercialisation, income generation, export markets and value-added product developments. However, there are limited literature on the handling, processing, shelf-life extension, and value-addition of the white sapote fruits produced in Ethiopia. The fruit's drying characteristics have not been studied and analysed well yet. Exceptional consideration must be paid to maintaining and improving the quality of this vital fruit. Hence, preservation techniques and technology advancements, including drying methods and pre-drying treatments, are required to prolong the shelf-life of white sapote fruits.

Drying is among the technologies regularly used for the preservation of fruits and vegetables, including white sapote fruit [6]. Drying methods such as sun-, oven- and solar-drying utilise heat to remove water from food by evaporation [6]. The open sun- and solar-drying rely on solar energy, whereas the oven method uses electricity [7,8]. Low-cost drying methods like solar- and open sun-dryers are feasible for smallholder farmers and have an economic advantage [9]. On the other hand, an oven dryer is suitable for quality fruit drying [10]. Therefore, the choice of drying method relies on the type of produce, technology accessibility, dryer cost, energy utilisation and source, dehydration cost, and the dried product's final quality [11].

Blanching is a crucial stage in fresh produce drying processing due to its numerous benefits, including the inactivation of the innate enzymes that are accountable for the discolouration of the end product [12]. Conventional hot water blanching inactivates undesirable enzymatic responses, killing the microorganisms, losing the hardness/texture, and easing drying [13]. Sulﬁtation has been extensively used in fruit and vegetable products manufacturing to decrease blackening throughout the drying process and hinder quality damage throughout the postharvest handling and storage period [13,14]. Sulfuring is frequently executed with sulfur dioxide gas or water-soluble sulﬁde salts, including potassium metabisulﬁte (K_2_S_2_O_5_), sodium metabisulﬁte (Na_2_S_2_O_5_) and sodium hydrogen sulﬁte (NaHSO_3_) [14]. Soaking plant tissue in NaCl solution before drying affects the behaviour of dehydrated material [15].

Drying kinetics is often presented by measuring the average product moisture content vs drying time [16,17]. During the initial drying stages, excess moisture on the product surface results in a rapid removal rate [18,19]. Subsequent drying of the material depends on the product-dependent rate at which internal water migrates to the product surface via diffusion [18,19]. Therefore, this study investigated the influence of drying methods and pre-drying treatments on the drying characteristics and drying models of white sapote slices.

## 2 Materials and methods

### 2.1 Collection and sampling of study material

Fully ripened white sapote (*Casimiroa edulis*) fruit samples were obtained and purchased from Bate kebele farmlands in Haramaya Woreda, East Hararghe Zone, Oromia Regional State. Matured fruits that were close to orange in colour with no blemishes and signs of disease on the skin were selected. The samples were collected from the tree early in the morning before sunrise and were transported, cleaned, washed and stored at 8 °C in a refrigerator until used for the experiment [20].

### 2.2 Sample Preparation

White sapote fruits were harvested, washed thoroughly with tap water, peeled with a stainless-steel knife and sliced into 2 mm thickness [21]. A 0.5 kg of slices of white sapote were weighed for each treatment and immersed in 0.25 % Sodium metabisulphite (Na_2_S_2_O_5_) [19] for 5 min at room temperature and dipped in 10 % Sodium chloride (NaCl) for 1 h at room temperature [20]. The other portion was blanched at 70 °C for 3 min [22] and immersed in pure distilled water to serve as a control.

### 2.3 Experimental plan

The experimental design was planned with two factors: three drying methods (oven, solar and sun drying) and four pre-drying treatments (P1, P2, P3, P4) (Table 1). The pre-drying treatments were treated by dipping in 0.25 % Sodium metabisulphite (Na_2_S_2_O_5_) solution for 5 min (P1) [22], dipping in 10 % Sodium chloride (NaCl) solution for 1 h at 25 °C (P2) [21], dipping in hot water at 70 °C for 3 min (P3) [23] and dipping in distilled water used as control (P4). The treatments were done in three replications.

**Table 1:** Experimental layout.

Factor 1 Drying Method (D)	Factor 2	Pretreatment (P)		
	P1	P2	P3	P4
D1	D1P1	D1P2	D1P3	D1P4
D2	D2P1	D2P2	D2P3	D2P4
D3	D3P1	D3P2	D3P3	D3P4

Where: D = Drying method; P = Pre retreatment; D1 = Oven drying; D2 = Solar drying; D3 = Sun-drying; P1 = Blanching; P2 = 0.25 % Sodium metabisulphite (Na_2_S_2_O_5;_ P3 = 10%Sodium chloride; and P4 = Control.

### 2.4 Drying of white sapote slices

#### 2.4.1 Oven drying

The sliced and pretreated white sapote was dried at a preset temperature of 65 °C for the laboratory-scale batch-type hot air oven model WHL-25AB made in the USA. The drying process was continued for 8 h [24]. The sample was weighed frequently every 15 min for the first 2 h of drying. Then, the time interval for taking the sample weight was extended to 30 min for the next 2 h of drying, and finally, the time gap was kept at 1 h [24].

#### 2.4.2 Solar drying

**Fig. 1:**
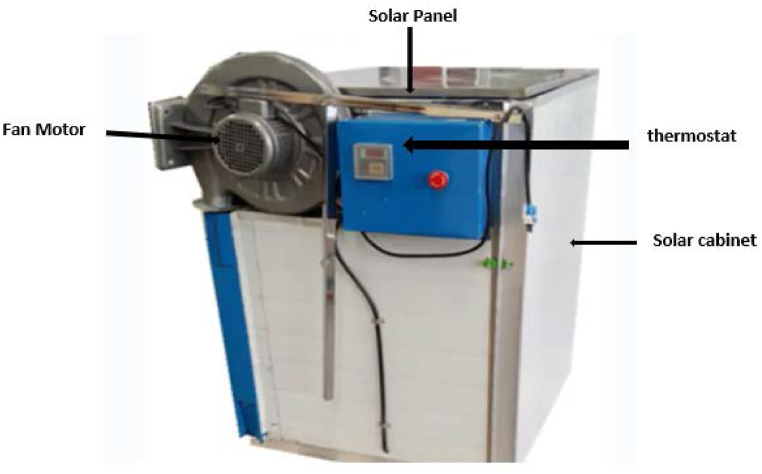
Solar dryer used for drying the white sapote slices during the experimentation.

**Fig. 2:**
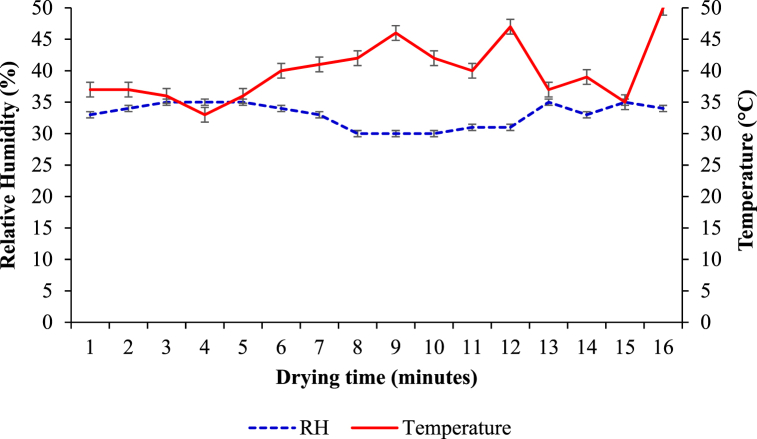
Variation of temperature and relative humidity during solar cabinet drying white sapote slices.

The other 0.5 kg treated slice samples were organised on the cabinet and loaded into the stainless-steel solar dryer of dimension 1 m*0.8 m*1.2 m (L*W*H) (model JX-1, and made in China) (Fig. 1). Moisture loss was documented every 15-min intervals for the first 2 h of drying. Then, the time gap increased to 30 min for the next 2 h' break. Finally, the drying time gap was kept every hour for the remaining drying period. Then, the weight of the sample was taken and immediately returned to the dryers for each drying time gap. The overall procedure of opening the door, taking samples from dryers, weighing them, and closing the door took only a minute. The overall drying process took 8 h [24]. The sample weight was measured precisely by an electronic balance with an accuracy level of 0.001g. The thermometer and a hygrometer were used to frequently measure drying air temperature and relative humidity in the drying cabinet, respectively. Drying air temperature observations of the temperature inside the drying cabinet ranged from 33 to 50 °C, and the relative humidity inside the chamber was measured to be 30–35 % (Fig. 2).

#### 2.4.3 Sun drying

**Fig. 3:**
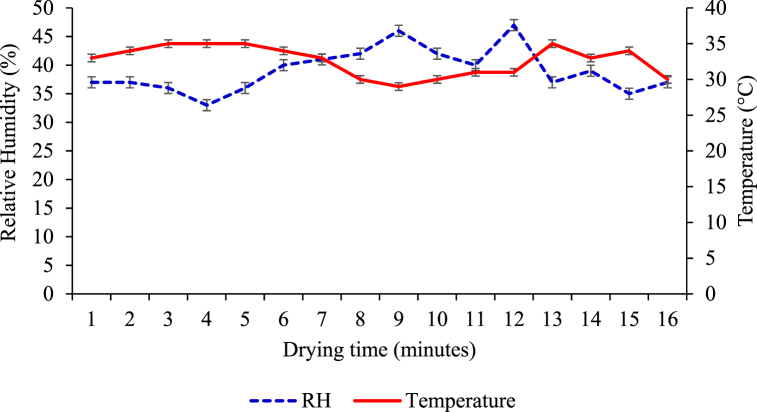
Variation of temperature and relative humidity during open sun drying white sapote slices.

Moisture loss was documented at each 15-min interval for the first 2 h of drying. Then, the interval increased to 30 min for the next 2 h, followed by an hour interval for the rest of the drying time. A slice of white sapote spreads on a black polythene sheet, drying under the open sun. The thermometer and a hygrometer were used to frequently measure drying air temperature and relative humidity on the open-sun polyethene drying sheets, respectively. Drying was continued until the sample attained a constant weight. Drying air temperature measurements on black polythene sheets varied from 29 to 35 °C, and the relative humidity of the ambient air was measured to be 33–47 % of the drying parameter estimation (Fig. 3).

#### 2.4.4 Moisture content

The moisture content of the fresh sapote slices was computed using the oven method [25]. Two grams of white sapote sample were weighed into a clean and dried crucible. The crucible and the sample were transferred into an oven and dried at 100 °C until a constant weight was obtained. The sample was cooled inside the desiccator, and the dried weight of the sample plus the crucible was noted. Moisture content percentage was calculated as follows (Eq. (1)):

(1) $$M\left(\text{db}\right)=\frac{\;\text{Mw}}{\text{Md}\;}\;X100$$

Where:

M (db) = Moisture content (% dry basis);

Mw = Mass of water (g); and.

Md = Mass of the dried sample (g).

#### 2.4.5 Moisture ratio

Moisture ratio (MR) is the moisture content ratio at any given time to the initial moisture content (comparative to the equilibrium moisture content). It was calculated using the method described by Ozbek and Dadali [26] (Eq. (2)).

(2) $$\text{MR}=\frac{M-\text{Me}}{Mo-Me}$$

Where:

M = instantaneous moisture content (%db);

Mo = initial moisture content (%db); and.

Me = equilibrium moisture content (EMC) of material (%db)

It should be noted that due to the insignificant value of Me in comparison with M and Mo, it can be saved; therefore, Eq. (2) can be simplified to Eq. (3) [15].

(3) $$\text{MR}=\frac{M}{Mo}$$

#### 2.4.6 Drying rate

Examination of drying kinetic provides the behaviour of food products beneath drying situations. The product's drying rate and moisture ratio under drying situations were used to understand the moisture elimination rate. The drying rate of white sapote slices was calculated [27], as Eq. (4):

(4) $$R=\frac{\text{Wr}}{\text{Tx}\;\text{Wd}\;}$$

Where:

R = Drying rate (g/minute);

Wr = amount of moisture removed (g);

T = Time taken (minutes); and.

Wd = Total bone-dry weight of the sample (g). Bone dry weight is the final constant weight a hygroscopic material reaches when completely dried out, and the product's weight is without moisture.

### 2.5 Drying models

Drying plots were fitted through four moisture ratio models: the Lewis, Simple Exponential, Page, and Henderson and Pabis models (Table 2). These models were used to determine the moisture ratio with drying period throughout the thin-layer drying approach that might corroborate with the drying of white sapote slices [28].

**Table 2:** Typical models for drying solids.

Model name	Model Equation
Lewis model	MR = exp^(−kt)^
Exponential model	MR = ae-^kt^
Page model	MR = exp (-^kt^n^)
Henderson and Pabis	MR = a exp (-kt^n)

Where:

MR is the moisture ratio,

k, a, n are constants in the model, and

*t* is time in minutes.

To select the best fit model to define drying behaviour accurately, the coefficient of determination (R^2^) (Eq. (5)) and the goodness of the fit were determined additionally using other numerical parameters, including root mean square error (RMSE*)* (Eq. (6)) and reduced chi-square (χ^2^) values (Eq. (7)).

(5) $$R^{2}=\frac{\sum_{i=1}^{N}MRprei-\;\bar{M}Rexpi}{{\left(MRprei-\;\bar{M}Rexpi\right)}^{2}}$$

(6) $$RMSE={\left[\frac{1}{N}\sum_{i=1}^{N}{\left(MRexpi-MRprei\right)}^{2}\right]}^{1/2}$$

(7) χ2=∑i=1N(MRexpi−MRprei)^2N−z

Where:

N is the total number of observations,

Z is the number of model parameters,

*MR*expi is the experimental moisture ratio value and.

*MRprei* is the predicted moisture ratio value.

### 2.6 Data analysis

Each treatment and measurement was carried out in triplicate. The goodness of fit of the tested mathematical models against the experimental values was assessed using the coefficient of determination (R^2^) and the reduced chi-square (χ^2^) values. The higher the R^2^ values and the lower the χ^2^ values, the better the goodness of fit [29]. The model parameters a and n (empirical coefficients) and k (the drying rate coefficient) were determined. Their combinations were optimised to suit the minimum value of the sum of square deviation (RMSE) on the expected data values (model values). The Excel Solver data tool was used for the optimisation and the final values of these constants.

## 3 Results and discussion

### 3.1 Drying characteristics of treated white sapote slices

#### 3.1.1 Hot air oven drying

**Fig. 4:**
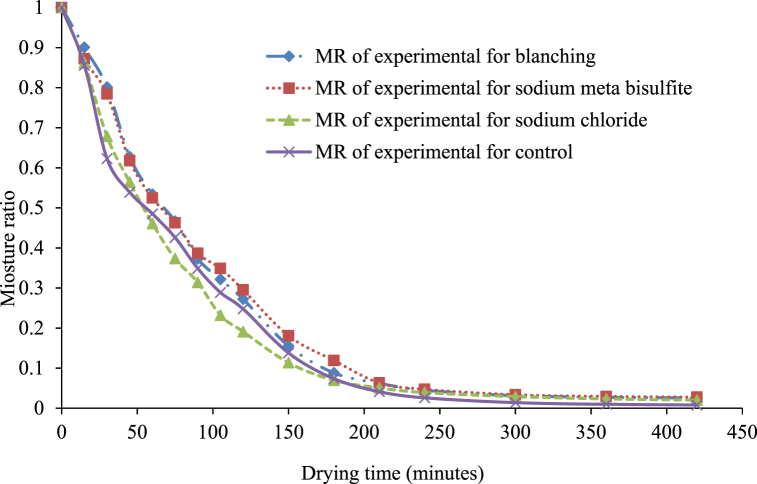
Moisture ratio versus drying time curves for white sapote slices dried in a hot air oven for different pretreatments.

The moisture ratio (MR) of treated and untreated white sapote slices reduced constantly with increasing drying period (Fig. 4) for oven drying. The drying rates for treated and untreated white sapote slice samples were initially elevated due to the high moisture content, but reduced rapidly to almost the same rate during drying. It was due to the availability of free water in blanched white sapote slice samples, which might have occurred because the slices absorbed more water during blanching. Agarry et al. [30] observed similar findings when they studied the thin-layer drying kinetics of pineapple influence of blanching temperature time-combination.

**Fig. 5:**
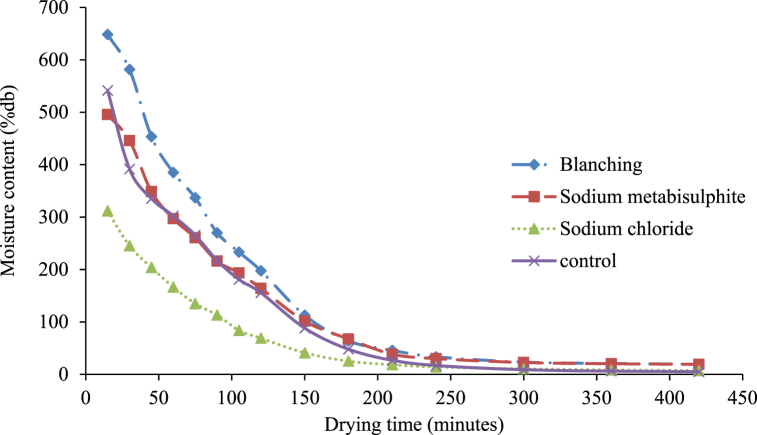
Drying characteristics of white sapote slices dried in hot air oven drying are different pretreatments.

Moreover, for the drying kinetic investigation, the quantity of water removed from samples as against drying time was represented as moisture content for oven drying. Fig. 5 shows that most of the moisture was removed in the early hours of drying in all the pretreatments. The high initial moisture content for blanched white sapote samples might have happened due to the slices gaining more moisture during blanching. Similar findings were observed by Agarry et al. [30], who studied the thin layer drying kinetics of pineapple influence of blanching temperature time-combination. It indicates that there is an effect of different pre-dring treatment applications on the drying features of white sapote slices. The study is aligned with Arun and Vishwavidyalaya [24] findings, who stated that the variation in the final moisture content of bananas difference happened due to the pretreatments used.

**Fig. 6:**
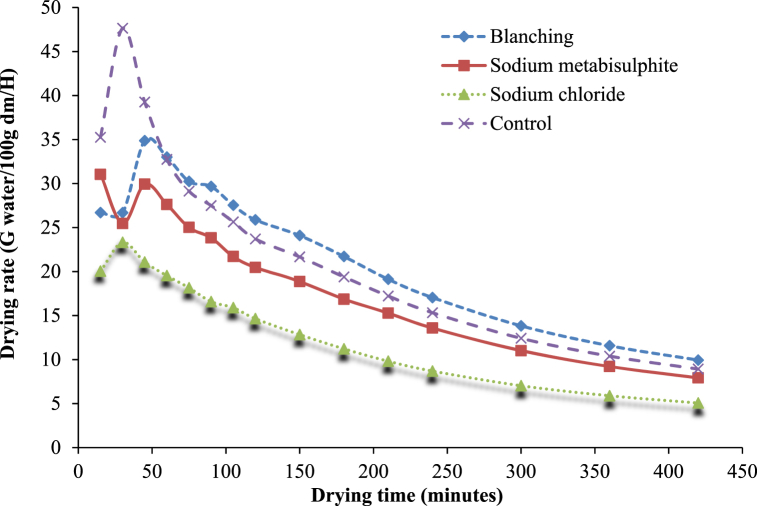
Drying rate versus time curves for white sapote slices dried in a hot air oven drying for different pretreatments.

Finally, in the drying kinetic investigation, the amount of water removed from samples against drying time was represented as the drying rate (g of water/100g of dm/h) for oven drying. Variations in drying rate (g of water/100g of dm/h) against drying time under diverse pre-drying treatments are shown in Fig. 6. It is evident from Fig. 6 that the whole drying procedure followed the falling rate period of drying, which is similar to other biological materials and is a natural phenomenon in these materials [31]. Furthermore, it can be seen that the drying rate was elevated at the early drying steps, which slowly reduced with the advancement of drying time. At the last drying stages, the drying rate was considerably less than that of the initial stage. There was no significant effect of pre-drying treatment on the drying rate of white sapote slices for oven drying. However, the drying rates of sodium chloride-treated white sapote slice samples at later drying stages were less than the other treated samples. This was because of the high resistance existing on the slices' surface, which was formed by sodium chloride, compared to other pretreatments. Alike results were observed by Vasconcelos et al. [32] on the effect of diverse chemicals on drying features during the drying of banana slices in untreated and treated cases.

#### 3.1.2 Solar drying

**Fig. 7:**
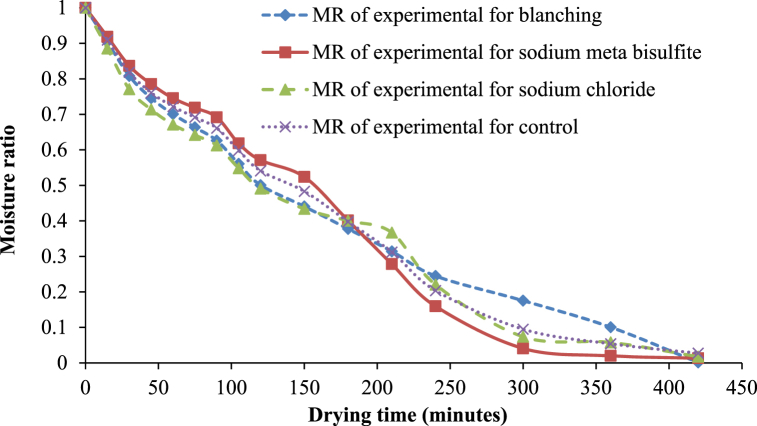
Moisture ratio curve of white sapote slices dried by solar dryer combined with different pretreatments.

Changes in the moisture ratio (MR) as a function of drying time and different pretreatments for solar drying are presented in Fig. 7. The MR value of treated and untreated white sapote slices decreased continuously with increasing drying time (Fig. 4) for solar drying. This may be similar with the findings of Agarry et al. [30] and Dikbasan [33] for the studies on the drying properties of treated pineapple and apple, respectively. The drying rates for treated and untreated white sapote slice samples initially rose due to the high moisture content. Still, they reduced quickly to nearly the same rate throughout the drying period. The drying kinetics behaviour recommends that the drying development happens mostly throughout the falling rate period is that has been informed for different agricultural commodiets [[34], [35], [36]].

**Fig. 8:**
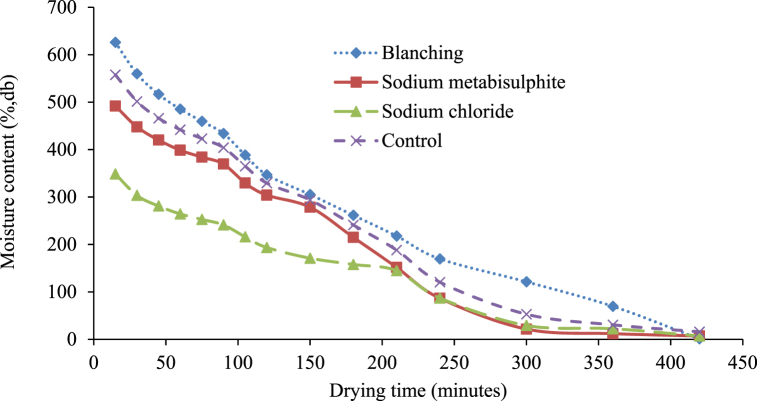
Drying characteristics of white sapote slices dried inside a solar drier with different pretreatments.

Furthermore, in the drying kinetic study, the quantity of water lost from samples against drying time was represented as moisture content for solar drying, as displayed in Fig. 8. The high initial moisture content for blanched white sapote samples perhaps happened due to the slices absorbing more water during blanching. Lower moisture content in the sample treated with sodium chloride probably occurred because of the slice loss due to osmotic behaviour in sodium chloride. Comparable results were observed by Agarry et al. [30], who studied the thin-layer drying kinetics of pineapple influence of combined blanching temperature and time. It is also due to the hindrance existing on the external of the slices, which was formed by sodium chloride [37].

**Fig. 9:**
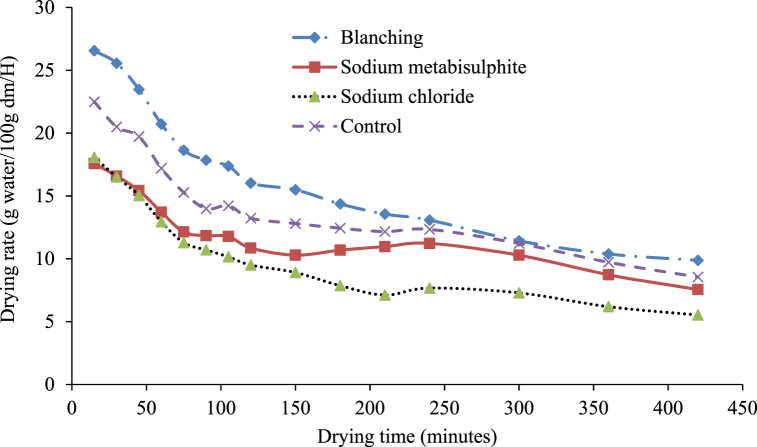
Drying rate curve of white sapote slices dried by solar dryer combined with different pretreatments.

Finally, in the drying kinetic investigation, the extent of water loss from samples against drying time was denoted as the drying rate (g of water/100g of dm/h) for solar drying, as shown in Fig. 9. It is obvious from the plot that the maximum drying rate was found in the first hour. Overall, the drying rate reduced as the drying period advanced, showing an inverse association between the drying rate and drying time. Deng et al. [13] were observed similar findings on the influence of various drying characteristics during banana slices drying. The lack of a constant rate period of drying recommends that no free water be available on the surface of the slices.

#### 3.1.3 Sun drying

**Fig. 10:**
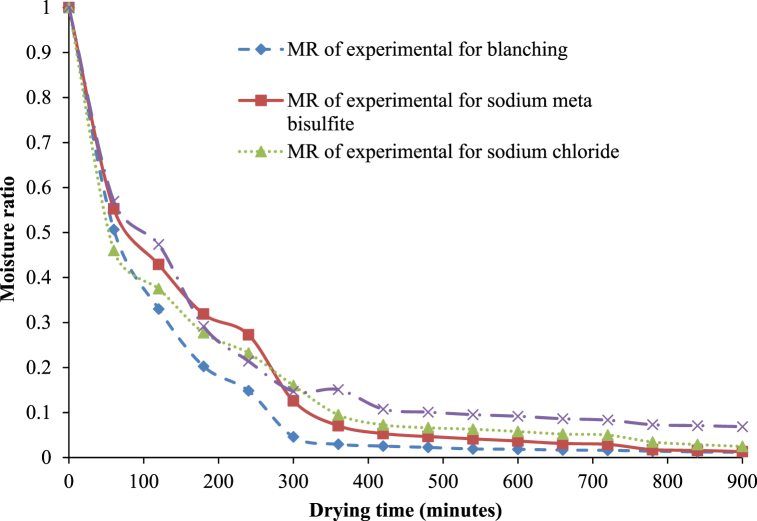
Moisture ratio versus drying time curves for white sapote slices dried under the open-sun combined with different pretreatments.

There is a nonlinear relationship between the moisture ratio and drying time, with a large decrease at the beginning, followed by a falling rate period at all treatments during the drying of white sapote slices (Fig. 10). This agrees with Agarry et al. [30] and Dikbasan [33] studies on theated pineapple and apple drying characteristics. Initially, the drying rates for treated and untreated white sapote slice samples were high because the moisture content was elevated but reduced quickly to almost the same rate during the drying period. The drying control samples were highly removed water during the drying process compared to the other treated samples in contrast with an oven and solar drying. This was due to the greater availability of free water in the control sample. A similar tendency was observed in the influence of various chemical pre-drying treatments on the drying feature of banana slices in the case of untreated and treated samples reported by Arun and Vishwavidyalaya [24].

**Fig. 11:**
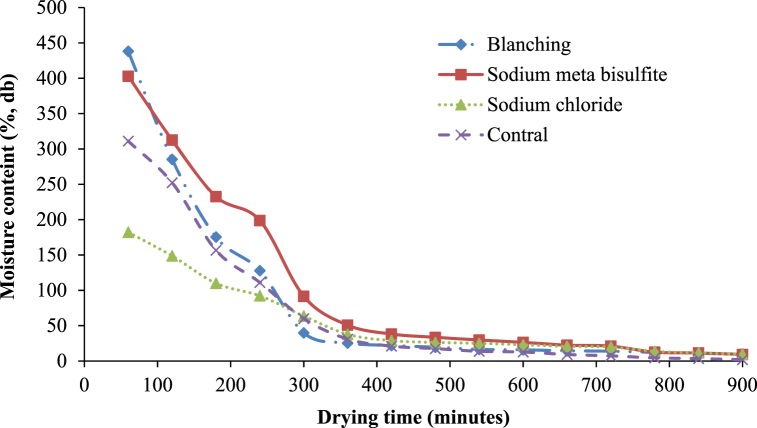
Drying characteristics of white sun slices dried in sun-drying with different pretreatments.

In the drying kinetic study, the extent of water dehydrated from samples against the drying time was represented by the open-sun drying in Fig. 11. It can be seen that the elimination at the early drying phase was high in all pre-drying treatments. However, the sample's final moisture content differed slightly (Fig. 11). The high initial moisture content for blanched white sapote samples might be due to the slices absorbing more moisture during blanching, and the final moisture content was lesser in the control sample than in the treated sample. Due to chemical treatment, there were probably some variations in the samples' textural charateristics. It was due to more availability of free water in control and the resistance excisted on the surface of the slices, which was formed by the pretreatments, *i.e.,* blanching, sodium Metabisulfite, and sodium chloride [37]. This may have occurred because of the gelatinisation of carbohydrates for the blanched white sapote sample [30]. The drying characteristics of the white sapote slice results initiated in this study were in close agreement with the reported drying characteristics of the banana slice [24].

**Fig. 12:**
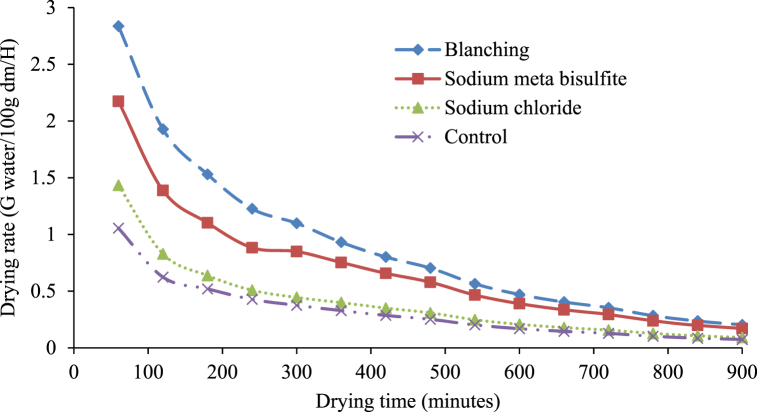
Drying rate curves for white sapote slices dried under the open sun combined with different pretreatments.

Finally, in the drying kinetic exploration, the quantity of water lost from samples against drying time, as the drying rate (g of water/100g of dm/h) for open-sun drying, was displayed in Fig. 12. The drying rate changes (g water/100g of dm/h) with drying time beneath diverse pre-drying treatments, as shown in Fig. 12. The whole drying process followed the falling rate period of drying due to the different cell arrangements and water activity in various food materials [38]. Furthermore, it can be seen that in the early drying stages, the drying rate advanced and was progressively reduced with the development of the drying period. The absence of a constant rate period of drying suggests that no free water was available on the slices' surface, as Abraham-Juarez et al. [39] and Agarry et al. [30] reported.

### 3.2 Drying models

Moisture ratio data of dried white sapote slices were fitted into thin-layer drying models listed in Table 3, Table 4, Table 5, Table 6. The Excel Solver data tool was used for the optimisation, and the final values of these constants are listed in Table 3, Table 4, Table 5, Table 6 The model parameters **a** and **n**, the empirical constants, and k, the drying rate constants, were determined. Their combinations were optimised to suit the requirements of the minimal value of the sum of square deviation on the expected data values (model values). For all the equations, the R^2^ values were greater than 0.95, indicating a good fit.

**Table 3:** Lewis model drying characteristics of sliced white sapote fruit.

Drying method	Pretreatments	RMSE	chi-square (χ^2^)	Constants(k)	R^2^
Oven drying	Blanching	0.00762541	0.000477	0.01112180	0.994623
	NaS_2_O_5_	0.009118	0.00057	0.010616	0.994348
	NaCl	0.004419	0.000276	0.013258	0.996837
	Control	0.011633	0.000727	0.012542	0.988447
Solar drying	Blanching	0.012805	0.0008	0.005768	0.98828
	NaS_2_O_5_	0.081768	0.005111	0.005606	0.961749
	NaCl	0.036538	0.002284	0.006045	0.965765
	Control	0.034555	0.00216	0.005632	0.977323
Sun drying	Blanching	0.007826	0.000489	0.009453	0.986924
	NaS_2_O_5_	0.021662	0.001354	0.006805	0.969956
	NaCl	0.054076	0.00338	0.007518	0.95474
	Control	0.052336	0.003271	0.006189	0.983983

**Table 4:** Exponential model drying characteristics of sliced white sapote fruit.

Drying method	Pretreatments	RMSE	chi-square (χ^2^)	Constants		R^2^
				k	a	
Oven drying	Blanching	0.01166	0.000729	0.011504	1.05293	0.99481
	NaS_2_O_5_	0.007572	0.000473	0.010961	1.027996	0.99471
	NaCl	0.003362	0.00021	0.013619	1.024716	0.99761
	Control	0.011605	0.000725	0.005714	0.99337	0.98853
Solar drying	Blanching	0.012677	0.000792	0.005714	0.99337	0.98873
	NaS_2_O_5_	0.074692	0.004668	0.005963	1.048188	0.95678
	NaCl	0.034894	0.002181	0.005845	0.976091	0.96820
	Control	0.033588	0.002099	0.005771	1.017956	0.97569
Sun drying	Blanching	0.00731	0.000457	0.009253	0.978276	0.98815
	NaS_2_O_5_	0.018845	0.001178	0.00647	0.951913	0.97405
	NaCl	0.045334	0.002833	0.006725	0.910699	0.97071
	Control	0.046246	0.00289	0.005662	0.927946	0.97982

**Table 5:** Page model drying characteristics of sliced white sapote fruit.

Drying method	Pretreatments	RMSE	chi-square (χ^2^)	Constants		R^2^
				k	n	
Oven drying	Blanching	0.004322	0.00027	0.004273	1.206207	0.99669
	NaS_2_O_5_	0.013677	0.000854	0.009449	1.030789	0.98713
	NaCl	0.025467	0.001592	0.009067	1.086815	0.99877
	Control	0.004444	0.000278	0.009067	1.086815	0.98438
Solar drying	Blanching	0.075855	0.004741	0.009374	1.078619	0.98709
	NaS_2_O_5_	0.024289	0.001518	0.00416	1.06441	0.99889
	NaCl	0.060496	0.003781	0.000734	1.401758	0.97088
	Control	0.059976	0.003749	0.004747	1.047203	0.97416
Sun drying	Blanching	0.041569	0.002598	0.001967	1.209621	0.98406
	NaS_2_O_5_	0.008473	0.00053	0.020624	0.83974	0.98958
	NaCl	0.016022	0.001001	0.018936	0.807495	0.97684
	Control	0.009764	0.00061	0.053789	0.620839	0.97061

**Table 6:** Henderson and Pabis model drying characteristics of sliced white sapote fruit.

Drying method	Pretreatments	RMSE	Chi-square	Constants			R^2^	
			(χ2)	a	k	n		
Oven drying	Blanching	0.002041	0.000255	1.029066	0.00553	1.154219	0.9974	
	NaS2O5	0.002496	0.000312	0.991842	0.005949	1.12435	0.9968	
	NaCl	0.001058	0.000132	1.005502	0.009443	1.07892	0.9988	
	Control	0.005793	0.000724	0.99351	0.011947	1.009225	0.9923	
Solar drying	Blanching	0.005399	0.000675	0.966262	0.003328	1.099239	0.992	
	NaS2O5	0.01281	0.001602	0.918333	0.000158	1.675755	0.9847	
	NaCl	0.001613	0.002017	0.941395	0.00291	1.127789	0.976	
	Control	0.00837	0.001047	0.938021	0.000883	1.34614	0.9886	
Sun drying	Blanching	0.00183	0.000229	0.93809	0.020516	0.84288	0.9966	
	NaS2O5	0.00579	0.000724	0.991791	0.056139	0.815281	0.9896	
	NaCl	0.003111	0.000389	0.996299	0.040181	0.613826	0.9874	
	Control	0.00633	0.000792	1.005022	0.000158	0.648918	0.9874	

The Lewis model χ^2^ values ranged from 0.00027 to 0.0.00072, 0.0008 to 0.0051 and 0.00049 to 0.0033 for the oven, solar and open-sun drying, respectively. For the exponential model, χ^2^ values ranged from 0.00021 to 0.00073, 0.00079 to 0.0047 and 0.00046 to 0.0082 for the oven, solar and sun drying, respectively. For the page model, the χ^2^values were 0.00027–0.0016, 0.0015 to 0.0047, and 0.00053 to 0.0026 for the oven, solar, and sun drying, respectively, and the Henderson and Pabis model, the χ^2^ ranged from 0.000132 to 0.000724, 0.00022918 to 0.0022017, 0.000389 to 000792 for the oven, solar and sun drying, respectively. The Lewis model R^2^ values ranged from 0.988 to 0.996, 0.961 to 0.988 and 0.955 to 0.987 for the oven, solar and sun drying, respectively. Moreover, for the exponential model, R^2^ values ranged from 0.988 to 0.997, 0.956 to 0.988, and 0.971 to 0.988 for the oven, solar, and sun drying, respectively. The Page model R^2^ value ranged from 0.995 to 0.997, 0.971 to 0.998 and 0.971 to 0.989 for the oven, solar and sun drying, respectively and Henderson and Pabis model R^2^ value ranged from 0.99768 to 0.9988, 0.9760 to 0.9923, 0.9874 to 0.9936 for the oven, solar and sun drying, respectively which can could sufficiently define the drying characteristics of the sliced white sapote in thin layers.

Henderson and Pabis's model has been reported as the best fit to describe and predict the drying behaviour of thin-layered drying processes [33]. The Henderson and Pabis model's highest R^2^ (0.999) and lowest χ^2^ (0.000132) were recorded for the white sapote slice treated with sodium chloride to the pretreatments. This indicates that the drying method and pretreatment are the factors most affecting the drying rate during the dehydration of the white sapote slice.

## 4 Conclusions

The drying behaviour of white sapote slices of different pretreatments for oven drying, solar drying, and sun drying was the plot of moisture ratio versus drying time, moisture content (% db) versus drying time, and drying rate versus drying time. In the initial drying stages, the drying rate was higher, which gradually reduced with the progress of drying time. At the final drying stages, the drying rate was much lower than the initial stage. Drying curves were fitted with four moisture ratio models: the Lewis model, the simple exponential model, the Page model, and the Henderson and Pabis model. The Excel Solver data tool was used for the optimisation and the final values. The model parameters **a** and **n**, which are the empirical constants and k, and the drying rate constants were determined. Their combinations were optimised to suit the requirements of the minimal value of the sum of square deviation on the expected data values (model values). It can be concluded that the Henderson and Pabis model for the oven is due to drying techniques, and the Page model for Sodium metabisulphite due to pretreatments might well describe the drying characteristics of the white sapote fruit slices. The present result suggests that the Henderson and Pabis model for oven due to drying techniques and the Page model for Sodium metabisulphite due to pretreatments describe the drying characteristics of the white sapote fruit slices.

## Data availability statement

Data included in article/supp material/referenced in this article can be attached as a supplementary Excel file.

## CRediT authorship contribution statement

**Tariku Workneh Daksa:** Writing – review & editing, Writing – original draft, Visualization, Validation, Methodology, Investigation, Formal analysis, Data curation, Conceptualization. **Getachew Neme Tolesa:** Writing – review & editing, Writing – original draft, Validation, Supervision, Resources, Project administration, Methodology, Funding acquisition, Formal analysis, Data curation, Conceptualization.

## Declaration of competing interest

The authors declare the following financial interests/personal relationships which may be considered as potential competing interests:

Tariku Workneh Daksa reports financial support, administrative support, and equipment, drugs, or supplies were provided by The Ministry of Education (MoE) of the Federal Democratic Republic of Ethiopia and Haramaya University, Ethiopia. If there are other authors, they declare that they have no known competing financial interests or personal relationships that could have appeared to influence the work reported in this paper.
